# Diversity in Resource Use Strategies Promotes Productivity in Young Planted Tree Species Mixtures

**DOI:** 10.1111/gcb.70493

**Published:** 2025-09-26

**Authors:** Joel Jensen, Haben Blondeel, Joannès Guillemot, Florian Schnabel, Hernán Serrano‐León, Harald Auge, Lander Baeten, Nadia Barsoum, Jürgen Bauhus, Christel Baum, Raimundo Bermudez, Friderike Beyer, Pedro Brancalion, Jeannine Cavender‐Bares, Nico Eisenhauer, Adam Felton, Olga Ferlian, Sebastian Fiedler, Tobias Gebauer, Douglas L. Godbold, Peter Hajek, Jefferson S. Hall, Dirk Hölscher, Hervé Jactel, Holger Kreft, Cathleen Lapadat, Chloe MacLaren, Nicolas Martin‐StPaul, Céline Meredieu, Simone Mereu, Christian Messier, Rebecca A. Montgomery, Bart Muys, Charles A. Nock, John D. Parker, William C. Parker, Gustavo B. Paterno, Michael P. Perring, Quentin Ponette, Catherine Potvin, Peter B. Reich, James Rentch, Boris Rewald, Agnès Robin, Michael Scherer‐Lorenzen, Hans Sandén, Katherine Sinacore, Rachel J. Standish, Artur Stefanski, Kris Verheyen, Laura J. Williams, Martin Weih

**Affiliations:** ^1^ Department of Crop Production Ecology Swedish University of Agricultural Sciences Uppsala Sweden; ^2^ Department Environment, Forest and Nature Lab, Campus Gontrode Ghent University Melle‐Gontrode Belgium; ^3^ CIRAD. UMR Eco&Sols Montpellier France; ^4^ Eco&Sols, Univ Montpellier, CIRAD, INRAE, Institut Agro, IRD Montpellier France; ^5^ Forest Science Department University of São Paulo/ESALQ Piracicaba Brazil; ^6^ Chair of Silviculture, Faculty of Environment and Natural Resources University of Freiburg Freiburg Germany; ^7^ Geobotany, Faculty of Biology University of Freiburg Freiburg Germany; ^8^ Department of Community Ecology Helmholtz Centre for Environmental Research – UFZ Halle Germany; ^9^ German Centre for Integrative Biodiversity Research (iDiv) Halle‐Jena‐Leipzig Leipzig Germany; ^10^ Centre for Forest Management, Forest Research Alice Holt Lodge Farnham Surrey UK; ^11^ Soil Science, Faculty of Agriculture, Civil and Environmental Engineering University of Rostock Rostock Germany; ^12^ Department of Forest Resources University of Minnesota St. Paul Minnesota USA; ^13^ Center for Carbon Research on Tropical Agriculture University of São Paulo Piracicaba São Paulo Brazil; ^14^ re.green Rio de Janeiro Rio de Janeiro Brazil; ^15^ Department of Ecology, Evolution and Behavior University of Minnesota St. Paul Minnesota USA; ^16^ Institute of Biology Leipzig University Leipzig Germany; ^17^ Southern Swedish Forest Research Centre Swedish University of Agricultural Sciences Alnarp Sweden; ^18^ Department of Plant Ecology Technical University of Berlin Berlin Germany; ^19^ geo‐konzept society for environmental planning systems mbH Adelschlag Germany; ^20^ Department of Ecosystem Management, Climate and Biodiversity, Institute of Forest Ecology University of Natural Resources and Life Sciences (BOKU) Vienna Austria; ^21^ Department of Forest Protection and Wildlife Management Mendel University Brno Czech republic; ^22^ Smithsonian Tropical Research Institute, Forest GEO Panama City Panama; ^23^ Tropical Silviculture and Forest Ecology University of Göttingen Göttingen Germany; ^24^ University of Bordeaux, INRAE, BIOGECO Cestas France; ^25^ Biodiversity, Macroecology and Biogeography University of Göttingen Göttingen Germany; ^26^ INRAE, Ecologie des Forêts Méditerranéennes (URFM) Avignon France; ^27^ INRAE, BIOGECO Cestas France; ^28^ National Research Council‐Institute of Bioeconomy (CNR‐IBE) Sassari Italy; ^29^ National Biodiversity Future Center (NBFC) Palermo Italy; ^30^ Foundation Euro‐Mediterranean Center on Climate Change (CMCC) Sassari Italy; ^31^ Département des sciences biologiques Université du Québec à Montréal (UQAM) et en Outaouais (UQO) Montréal Canada; ^32^ Department Forest, Nature and Landscape KU Leuven Leuven Belgium; ^33^ Department of Renewable Resources University of Alberta Edmonton Alberta Canada; ^34^ Smithsonian Environmental Research Center Edgewater Maryland USA; ^35^ Ontario Ministry of Natural Resources Sault Ste. Marie Ontario Canada; ^36^ UK Centre for Ecology and Hydrology (UKCEH), Environment Centre Wales Bangor UK; ^37^ The UWA Institute of Agriculture The University of Western Australia Perth Western Australia Australia; ^38^ Université catholique de Louvain, Earth and Life Institute, Environmental Sciences Louvain‐la‐Neuve Belgium; ^39^ Department of Biology McGill University Montréal Québec Canada; ^40^ Smithsonian Tropical Research Institute Panama City Panama; ^41^ Institute for Global Change Biology University of Michigan Ann Arbor Michigan USA; ^42^ West Virginia University Morgantown West Virginia USA; ^43^ Smithsonian Tropical Research Institute ForestGEO, Agua Salud Project Panama City Panama; ^44^ USDA Forest Service, Human Dimensions Fort Collins Colorado USA; ^45^ School of Environmental and Conservation Sciences Murdoch University Murdoch Western Australia Australia; ^46^ College of Natural Resources University of Wisconsin Stevens Point Stevens Point Wisconsin USA; ^47^ Hawkesbury Institute for the Environment, Western Sydney University Penrith New South Wales Australia

**Keywords:** climate change mitigation, complementarity effects, forest management, functional traits, mixed‐species forest plantations, selection effects, tree species richness, TreeDivNet

## Abstract

Mixed‐species forestry is a promising approach to enhance productivity, increase carbon sequestration, and mitigate climate change. Diverse forests, composed of species with varying structures and functional trait profiles, may have higher functional and structural diversity, which are attributes relevant to a number of mechanisms that can influence productivity. However, it remains unclear whether the context‐dependent roles of functional identity, functional diversity, and structural diversity can lead to a generalized understanding of tree diversity effects on stand productivity. To address these gaps, we analyzed growth data from 83,600 trees from 89 species across 21 young tree diversity experiments spanning five continents and three biomes. Results revealed a positive saturating relationship between tree species richness and stand productivity, with reduced variability in growth rates among more diverse stands. Structural equation modeling demonstrated that functional diversity mediated the positive effects of species richness on productivity. We additionally report a negative relationship between structural diversity and productivity, which decreased with increasing species richness. When partitioning net diversity effects, we found that selection effects played a dominant role in driving the overall increase in productivity in these predominantly young stands, contributing 77% of the net diversity effect. Selection effects increased with diversity in wood density. Furthermore, acquisitive species with lower wood density and higher leaf nitrogen content had higher productivity in more diverse stands, while conservative species showed neutral to slightly negative responses to species mixing. Together, these results suggest that combining acquisitive with conservative species allows acquisitive species to drive positive selection effects while conservative species tolerate competition. Thus, contrasting resource‐use strategies can enhance productivity to optimize mixed‐species forestry, with potential for both ecological and economic benefits.

## Graphical abstract

By analyzing data from over 83,000 trees in 21 experiments across five continents, we found that forests with more species were more productive and had more stable growth. Productivity was driven mainly by fast‐growing species with acquisitive traits, while slower‐growing species coexisted without major losses. These results show that combining contrasting resource‐use strategies enhances forest productivity and supports mixed‐species forestry as a sustainable management approach.

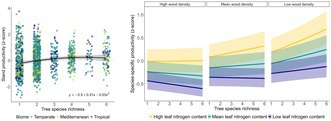

## 1 Introduction

The accelerating impacts of climate change require mitigation actions (Abbass et al. 2022). Forests can play a crucial role in mitigating climate change and its consequences through carbon sequestration. However, their capacity to serve as a carbon sink depends on their resilience to changing climatic conditions and associated environmental changes (IPCC 2021). Mixed‐species forests have been advocated for more than a century (Gayer 1886; Möller 1922); however, it took a long time to develop a scientific understanding of the mechanisms that distinguish them from monospecific forests (Bauhus et al. 2017). Now they are widely recognized as a promising approach to meeting these challenges (Blondeel et al. 2024; Depauw et al. 2024; Gamfeldt et al. 2013; Messier et al. 2019). Diverse forests are increasingly valued for potential benefits like enhanced productivity, carbon sequestration, resilience to disturbances, and improved human well‐being (Felton et al. 2024; Methorst et al. 2021; Rozario et al. 2024). Multiple studies indicate that diverse tree stands can surpass the average growth performance of monocultures (i.e., overyielding), contributing to both carbon sequestration and economic gains (Chamagne et al. 2017; Condés et al. 2023; Grossman et al. 2017; Messier et al. 2021). However, these findings are usually based on observational studies in mature forests, where diversity effects can be obscured by environmental heterogeneity (Bauhus et al. 2017; Pardos et al. 2021; Scherer‐Lorenzen et al. 2007). While controlled experiments manipulating tree species richness have provided critical insights, results have been variable due to different environmental and management conditions, ranging from negative to neutral to positive outcomes, and multi‐site experimental studies remain limited (Jucker et al. 2020; Sinacore et al. 2023; Tobner et al. 2016; Toïgo et al. 2022). Most broad geographical scale assessments are based on meta‐analyses of experimental studies (Jactel et al. 2018; Zhang et al. 2012), which account for between‐study variation to estimate overall effect sizes. These approaches are not sufficient to explore the mechanisms driving diversity effects within sites, thus leaving gaps in our understanding of global patterns.

As the number of species in a community increases, the potential for divergence in ecological niches and functional strategies, that is, functional diversity, also increases (MacArthur 1970; Turnbull et al. 2016). A greater diversity of ecological strategies enhances the likelihood that different species will utilize the same resource in distinct ways, potentially resulting in complementary resource use and therefore positive diversity‐productivity relationships (Belluau et al. 2021; Forrester and Bauhus 2016). Additionally, tree diversity can promote structural diversity, characterized by varied tree forms and complex canopy structures. Structural diversity can result both from distinct strategies among species and also from individual tree responses to neighbour effects, for example, crown plasticity (Jucker et al. 2015; Sapijanskas et al. 2014; Schnabel et al. 2019; Williams et al. 2020). Structural diversity is associated with more efficient canopy space occupation, which enhances light capture and/or reduces competition for light (Pretzsch 2014; Ray et al. 2023; Williams et al. 2017), which ultimately promotes forest growth (Ali et al. 2016; Forrester 2019; Sanaei et al. 2021). Both functional and structural diversity can therefore drive complementary species interactions and diversity–productivity relationships. Consequently, the historical preference of monocultures over mixtures in forestry may have reduced overall forest productivity, especially under disturbance regimes (Hlásny et al. 2021), potentially leading to lower carbon stocks in woody biomass (Bonan 2008). However, the selection of highly productive timber species in monocultures may have resulted in relatively high carbon stocks compared to mixed‐species stands. Despite these dynamics, the respective contributions of functional and structural diversity to forest growth at broad geographical scales have yet to be assessed under experimental conditions.

Another key question that remains is how strongly the functional identity of tree species in a community can influence ecosystem functioning as a whole. Partitioning the net effect of tree species diversification on stand productivity into selection and complementarity effects can elucidate the answer to this question. Selection effects quantify whether changes in growth are disproportionately driven by a few productive species, whereas complementarity effects quantify mean changes in growth across all species in a diverse stand (Loreau and Hector 2001). These effects can be positive and lead to overyielding, where a diverse mix is more productive than its respective monoculture, or they can be negative and lead to underyielding. Both the selection and complementarity effects can depend on the functional identities and functional diversity of species present, which jointly determine the species' growth potential and niche space available to do so. So far, research on grassland and tree communities has demonstrated that both the functional identity and diversity of communities are important for explaining diversity effects on aboveground biomass (Bongers et al. 2021; Nadrowski et al. 2010; Roscher et al. 2012; Tobner et al. 2016).

Ecological strategies of tree species are reflected in their functional traits. Identifying the trait profiles of species that perform well in diverse mixes and contribute to both selection and complementarity effects is important to understanding how biodiversity influences community productivity. Species diverge across a spectrum of resource‐use strategies, often categorized into conservative species (characterized by slow resource capture and efficient use) and acquisitive species (characterized by their rapid resource uptake) (Guillemot et al. 2022; Reich 2014). Based on these characteristics, acquisitive species tend to be more competitive than conservative species when grown under resource‐rich conditions, while conservative species often perform better than acquisitive species when grown under resource‐poor conditions (Lambers et al. 2008). Traits like wood density (WD), specific leaf area (SLA) and leaf nitrogen content (LNC) are useful in defining resource‐use strategies along an acquisitive‐conservative continuum (Díaz et al. 2016; Poorter et al. 2008; Poorter and Bongers 2006; Wright et al. 2003, 2004, 2010). On this continuum, species with low WD and high LNC and SLA indicate an acquisitive resource‐use strategy, while high WD and low LNC and SLA align with a more conservative strategy. More acquisitive species may be better equipped to exploit any additional resources facilitated by niche differentiation, suggesting potential to drive positive selection effects. In contrast, more conservative species may be more resilient to competition, minimizing negative impacts from species mixing and contributing to complementarity effects (Tobner et al. 2016). Understanding the mechanisms linking tree diversity to stand productivity, and how species with different functional identities perform in mixtures, will contribute to the development of biodiversity‐ecosystem function theory and help guide the design of beneficial species mixtures for forest managers, while also informing strategies to counteract the effects of biodiversity loss under global change.

Previous studies have investigated overyielding at either the species or community level by examining which species over‐ or underyield in mixtures (Zheng et al. 2024), or how community properties like functional identity or diversity lead to stand‐level over‐ or underyielding (Belluau et al. 2021; Bongers et al. 2021; Finegan et al. 2015). Our study brings these two angles of investigation together, leveraging raw data from global planted tree diversity experiments within the Tree Diversity Network (TreeDivNet; https://treedivnet.ugent.be/; Verheyen et al. 2016). Specifically, we hypothesize that (H1) aboveground stand productivity increases with tree species richness, and that (H2) these effects are mediated by positive effects of functional and structural diversity. Furthermore, we hypothesize that (H3) selection and complementarity effects depend on community trait composition, and that (H4) acquisitive species disproportionately benefit from species mixing compared to conservative species. By integrating species‐ and community‐level analyses from highly standardized biodiversity‐ecosystem function experiments, this study offers new insights into the mechanisms driving diversity‐productivity relationships and informs strategies for designing resilient and productive forests.

## 2 Materials and Methods

### 2.1 Study Sites and Data Collection

**FIGURE 1:**
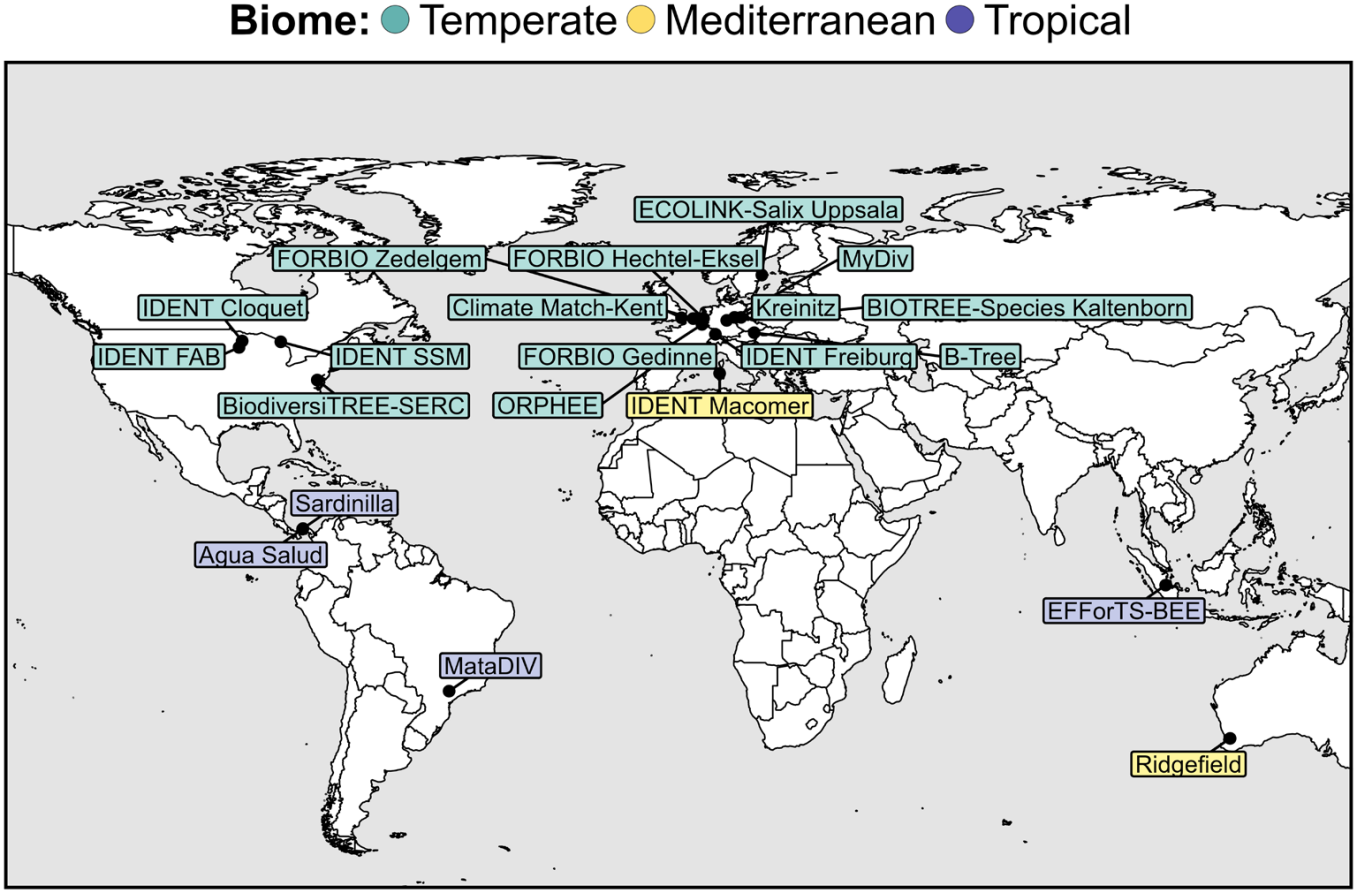
Locations of the 21 tree diversity experiments from the TreeDivNet network included in this study, colored by biome. Detailed information on each experimental site can be found in Table S1. Map lines delineate study areas and do not necessarily depict accepted national boundaries.

We compiled diameter and height data at the individual tree level from 21 experiments belonging to TreeDivNet (Paquette et al. 2018; Verheyen et al. 2016). The experiments are located in Europe, North and South America, Asia, and Oceania, spanning a diverse range of climatic conditions in temperate, Mediterranean, and tropical biomes (Figure 1; Table S1).

All experiments utilized a selection of locally‐adapted tree species suited to the site's climate and soil conditions. Species were planted both as monocultures and in mixtures with varying levels of species richness (i.e., count of species planted in a stand), with all trees within an experiment planted at the same time. A replicated randomized experimental design was used to distinguish between tree identity effects and tree diversity effects on forest functioning while controlling for environmental variability (Verheyen et al. 2016). The compiled dataset covers 83,600 individual trees across 1609 plots. The ages of the trees used in our analyses ranged between 4 and 16 years (average 9 years). To better capture species interactions at the most mature tree ages available, stem diameter and height data from the most recent available inventory year were used for each experiment (Table S1). Analyses included only plots where species diversity was the sole experimentally manipulated factor, excluding plots, or trees within subplots, with additional abiotic manipulations (e.g., fertilizer addition or irrigation). Analyses were restricted to the monoculture plots and the plots with a maximum richness of six species as only 2 out of 21 experiments exceeded this level. At the ECOLINK‐Salix Uppsala site, genetic diversity (i.e., *Salix* varieties) was used instead of taxonomic diversity. For multi‐stemmed trees, that is, trees with two or more stems growing from one root, the total diameter per tree was calculated as:

(1) $$\text{Diameter}=\sqrt{\sum_{i=1}^{n}\mathrm{ste}m_{i}^{2}}$$

### 2.2 Productivity Calculations

Stand productivity was defined as the annual basal area increment per hectare (m^2^ ha^−1^ year^−1^) at the plot level. Basal area, which is strongly correlated with woody biomass (Babst et al. 2014), was calculated for individual trees as:

(2) $${\text{Basal area}}_{\text{tree}}=\pi \times {\left(\text{diameter}/2\right)}^{2}$$

Annual productivity per plot was calculated by summing the basal areas of all individual trees within each plot, thereby accounting for the contribution of tree mortality. This value was then scaled to a per‐hectare basis and divided by the years since experiment establishment. For sites where inventories were completed on a subset of trees within each plot (Table S1), productivity was estimated by adjusting the total basal area of all measured trees in proportion to the sampled area before scaling to a per‐hectare basis. Details of specific experiment considerations are provided in Supporting Information.

### 2.3 Additive Partitioning

The net effect of diversity can be measured as the difference (in m^2^ ha^−1^ year^−1^) between the observed productivity of a mixed stand and its expected productivity based on the weighted mean productivity of its component species in monocultures. We calculated the expected productivity for a given species in a mixture by multiplying its proportion of the total tree count by its monoculture productivity (the total expected productivity of a mixture is the sum of each species' expected productivity). To separate the mechanisms by which species diversity enhances productivity, we partitioned the net diversity effect into the selection and complementarity effects (Loreau and Hector 2001). The selection effect was quantified as the covariance between species' monoculture productivity and the difference between their expected and observed productivity in a mixture, while the complementarity effect was calculated as the mean difference between expected and observed productivity for each species in the mixture.

### 2.4 Trait Data

We obtained species‐specific trait data from experiment‐specific measurements when possible (56 out of 267 values), and complemented these with published data where possible (187 out of 267 values), for example, mainly from the TRY Plant Trait Database (Kattge et al. 2011; https://www.try‐db.org/TryWeb/) (Table S2). We considered SLA (cm^2^ g^−1^), LNC (%) and WD (g cm^−3^) as key traits relating to plant function and resource use strategy (Poorter and Bongers 2006; Swenson and Enquist 2007; Westoby et al. 2002; Wright et al. 2010). We assessed the relation among species‐specific traits using principal component analysis (PCA) with the *rda* function in the *vegan* package (Oksanen et al. 2013). PC1 axis aligned with SLA and LNC explaining 56% of variation across traits, while PC2 aligned with WD explained 32% of trait variation (Figure S6). Given the high association between SLA and LNC (Figure S6), we opted to choose only one of these traits to represent the resource‐use strategy alongside WD in order to minimize redundancy. LNC was chosen because it also reflects nutrient allocation, capturing a more comprehensive aspect of resource use strategies, while both SLA and WD primarily reflect biomass allocation.

### 2.5 Functional and Structural Diversity

We quantified structural diversity within each plot using both the coefficient of variation (CV) and the Gini coefficient of individual tree heights (Gini 1912); both metrics showed similar correlations with species richness. We calculated functional diversity as well as the abundance‐weighted functional dispersion (FDis; Laliberté and Legendre 2010), which is calculated as the mean distance of each species to the centroid of a multidimensional trait space, weighing species distances by their relative abundance. We calculated FDis both for each specific trait separately (FDis_LNC_, FDis_WD_), as well as for all combined trait profiles (FDis_all_). We represented the stand's functional identity using separate community weighted means (CWM) for LNC and WD (CWM_LNC_, CWM_WD_), calculated as the average trait value per plot weighted by the number of individual trees per species within the plot.

### 2.6 Standardization

To minimize between‐experiment variability, all continuous variables that varied within experiments (i.e., stand productivity, CWM_LNC_, CWM_WD_, FDis_LNC_, FDis_WD_, FDis_all_, and structural diversity) were standardized within each experiment. Variables that did not vary within experiments (i.e., species‐specific trait values) were standardized across experiments. Functional dispersion metrics (i.e., FDis_LNC_, FDis_WD_, and FDis_all_) were standardized using min‐max scaling (i.e., rescaled between zero and one by subtracting the minimum value and dividing by the range), allowing for comparisons on a uniform scale while preserving the relative range. Other variables (i.e., stand productivity, CWM_LNC_, CWM_WD_, and structural diversity) were standardized using *z*‐score transformation (i.e., by subtracting the mean and dividing by the standard deviation). *z*‐Score standardization effectively sets each variable's unit as the number of standard deviations from the mean, retaining the relative distribution and central tendency within each experiment. The selection, complementarity, and net diversity effects were not transformed, as they were already centered relative to the constituent monocultures of each mixture. All standardizations were applied to the data subset corresponding to each specific analysis. A summary of all transformations for each variable is available in Table S3.

### 2.7 Statistical Analysis

To test our hypotheses, we fitted different mixed‐effects models using the *glmmTMB* package (Brooks et al. 2017), and structural equation modeling (SEM) using the *piecewiseSEM* package (Lefcheck 2016). All models assumed a normal (Gaussian) distribution of residual error. We accounted for the hierarchical data structure in all models with random effects (noted *u*_Random effect_) for species composition and block nested within experiment. Additionally, we included a random effect nested within experiment representing whether a species was classified as angiosperm or gymnosperm (“class”) to account for the different functional trait dynamics within these groups (Equation 8; Figure S1).

To examine the effect of species richness (1–6 species) on stand productivity (H1), we fitted a nonlinear mixed effects model incorporating a quadratic term. This term was statistically significant (*p* < 0.05) and improved model fit, as indicated by a reduction in AIC from 4055 to 4053. Biome and its interaction with species richness were initially included to account for potential variability across environments; however, neither term was significant and was subsequently excluded from the final model:

(3) $$\begin{array}{c} \text{Stand productivity}=\beta 0+\beta 1\times \text{species richness}+\beta 2\times {\text{species richness}}^{2}\\ +u_{\text{experiment}/\text{species composition}}+u_{\text{experiment}/\text{block}}+\epsilon \end{array}$$

To explore how functional and structural diversity mediates the relationship between species richness and stand productivity (H2), we developed three separate SEMs (Equations (4), (5), (6)). The first model (Equation 4; Figure S1) revealed a surprising negative relationship between structural diversity and stand productivity when interactions were excluded. This unexpected finding led us to explore potential underlying mechanisms. Consequently, we constructed a second model that incorporated an interaction between species richness and structural diversity (Equation 5; Figure 3a). This model contextualized and clarified the observed relationship by showing that the effects of structural diversity on stand productivity depended on species richness level. To delve deeper into the potential drivers of this interaction, we created a third model, replacing the species richness interaction with an interaction between functional and structural diversity (Equation 6; Figure S2). The SEMs were defined as follows:

(4) $$\begin{array}{c} \text{Structural diversity}=\beta 0+\beta 1\times \text{species richness}\\ +u_{\text{experiment}/\text{species composition}}+u_{\text{experiment}/\text{block}}+\epsilon 1 \end{array}$$

(5) $$\begin{array}{c} {\text{FDis}}_{\mathrm{all}}=\beta 2+\beta 3\times \text{species richness}\\ +u_{\text{experiment}/\text{species composition}}+u_{\text{experiment}/\text{block}}+\epsilon 2 \end{array}$$

(6) $$\begin{array}{c} \text{Stand productivity}=\beta 4+\beta 5\times \text{species richness}+\beta 6\times \text{structural diversity}\;\\ +\beta 7\times \text{FDisall}+\beta 8\times \left(\text{species richness}\times \text{structural diversity}\right)\;\\ +u_{\text{experiment}/\text{species composition}}+u_{\text{experiment}/\text{block}}+\epsilon 3 \end{array}$$

Structural equation modeling was done using data from 16 experiments for which the time interval between height and diameter measurements did not exceed 1 year. We tested the effect of structural diversity using both the CV and Gini coefficient. Both variables had comparable effects on stand productivity, but the Gini coefficient consistently improved model fit by AIC and was therefore used. Standardized path coefficients are shown for each pathway in the illustration of the SEM (Figure 3a), indicating how many standard deviations the response variables change for every 1 standard deviation change in the predictor.

To test how the composition of species in relation to stand functional identity and diversity in mixtures influenced the magnitude of selection, complementarity, and net diversity effects (H3), we employed linear mixed‐effects models. These models were used to examine the influence of functional identity and diversity on each diversity effect, with “diversity effect” used as a placeholder for the selection, complementarity, and net biodiversity effects:

(7) $$\begin{array}{c} \text{Diversity effect}=\beta 0+\beta 1\times {\mathrm{CWM}}_{\mathrm{WD}}+\beta 2\times {\mathrm{CWM}}_{\mathrm{LNC}}+\beta 3\times {\text{FDis}}_{\mathrm{WD}}\\ +\beta 4\times {\text{FDis}}_{\mathrm{LNC}}+u_{\text{experiment}/\text{species composition}}+u_{\text{experiment}/\text{block}}+\epsilon \end{array}$$

To investigate the effect of functional identity on species‐specific productivity in mixed stands (H4), we fitted a linear mixed‐effects model, including the interactions between species richness and each species‐specific functional trait:

(8) $$\begin{array}{c} \ln\left(\text{Species}‐\text{specific productivity}\right)=\beta 0+\beta 1\times \text{species richness}+\beta 2\times \mathrm{WD}\;\\ +\beta 3\times \mathrm{LNC}+\beta 4\times \left(\text{species richness}\times \mathrm{WD}\right)+\beta 5\times \left(\text{species richness}\times \mathrm{LNC}\right)\\ +u_{\text{experiment}/\text{species composition}}+u_{\text{experiment}/\text{block}}+u_{\text{experiment}/\text{class}}+\epsilon \end{array}$$

The model was adjusted to include only positive values by adding a small constant to each value, enabling a logarithmic transformation to meet the assumptions of residual normality. The values were back‐transformed for presentation in Section 3.

To examine changes in variability in productivity across species richness levels in H1, H3, and H4 models, we allowed variances to vary by tree species richness (*dispformula* argument in the *glmmTMB* package). We could then estimate residual variances in the dispersion models, which represent how the variability of outcomes changed in relation to species richness. This was not done for the models integrated within the SEM framework of H2, which required fixed residual variance structures to enable the derivation of *R* ^2^ values; a key requirement for evaluating model fit and explanatory power in SEMs.

Model performance was tested by checking residuals and diagnostics using the *performance* package (Lüdecke et al. 2021); testing for normality of residuals, multicollinearity, heteroscedasticity, and general model fit. The overall fit of the SEM was evaluated using AIC and Fisher's *C* statistic. All statistical analyses were conducted using R version 4.3.0 (R Core Team 2022).

## 3 Results

### 3.1 Increased Tree Diversity Enhances Aboveground Stand Productivity

**FIGURE 2:**
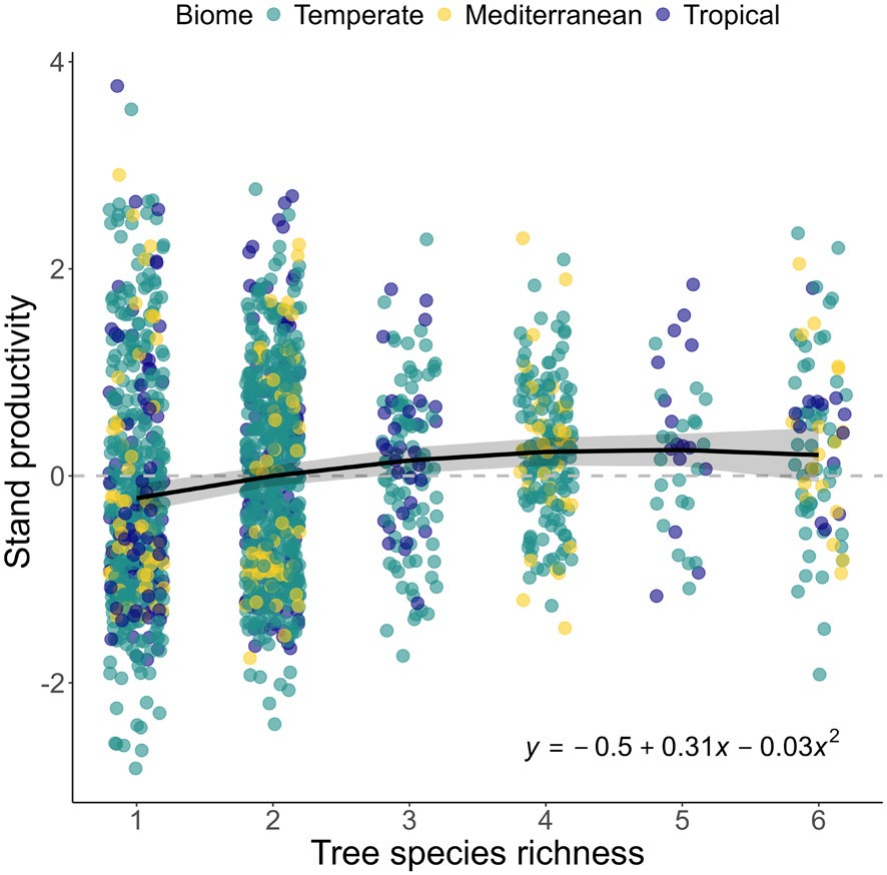
Relationship between stand productivity and species richness across 21 experiments and three biomes. Stand productivity corresponds to standardized annual basal area increment. Positive values indicate productivity levels above the average within each experiment. Absolute mean stand productivity (m^2^ ha^−1^ year^−1^) can be found in Table S3. Colors indicate biome. The dashed black line represents the mean stand productivity within each experiment. The solid black line represents the estimated mean productivity, with the shaded area showing a 95% confidence interval.

Stand productivity increased significantly with species richness (0.31 for the linear term, *p* < 0.01), with saturating effects at higher richness levels indicated by a negative quadratic term (−0.03, *p* < 0.05) (Figure 2; Table S4). Productivity peaked at four to five species, where it was 0.45–0.47 standard deviations higher than in monocultures. Additionally, variability in productivity within species richness levels decreased with increasing species richness, with residual variance decreasing by 0.03 for each additional species (*p* < 0.05, see Table S4), indicating a higher predictability of production in more diverse stands.

### 3.2 Functional but Not Structural Diversity Mediates Positive Species Richness Effects on Productivity

**FIGURE 3:**
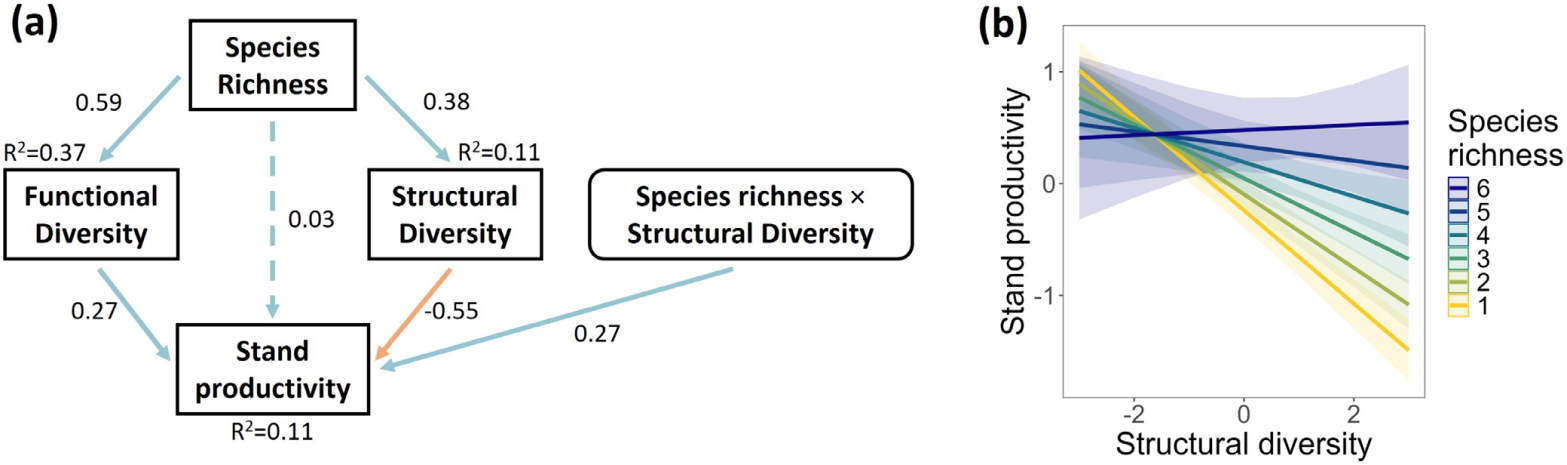
SEM illustrating direct and indirect links between (a) species richness and standardized stand productivity across 16 experiments (b) and the modeled interaction between species richness and structural diversity on stand productivity. The model displays standardized path coefficients for each pathway, and marginal *R* ^2^ values for each endogenous variable. Blue pathways indicate positive correlations, while orange pathways indicate negative correlations. Significant pathways (*p* < 0.05) are shown with solid lines. Individual pathways are modeled separately in Figure S3.

Species richness had both a positive indirect effect on stand productivity mediated through functional diversity (0.59 × 0.27 = 0.159) and a negative indirect effect on stand productivity mediated through structural diversity (0.38 × −0.55 = −0.209), obtained by multiplying the standardized path coefficients (Figure 3a). Stand productivity was also significantly affected by the interaction between species richness and structural diversity, with the influence of structural diversity becoming progressively less negative as species richness increased, getting slightly positive at the highest species richness level. This effect contributed an additional 0.27 standard deviations to stand productivity across the species richness gradient, so the effect of structural diversity on productivity turned positive after reaching five species (Figure 3b). The interaction between structural diversity and functional diversity, rather than species richness, as shown in Figure S2, also exhibited a positive effect. This interaction contributed an additional 0.19 standard deviations to stand productivity across the range of functional diversity. The direct effect of species richness on stand productivity (0.03) was not significant. The SEM is an adequate fit to the data based on the Fisher's *C* statistic (*C* = 1.063, *p* = 0.59).

### 3.3 Effects of Functional Identity and Diversity on Selection and Complementarity Effects

**FIGURE 4:**
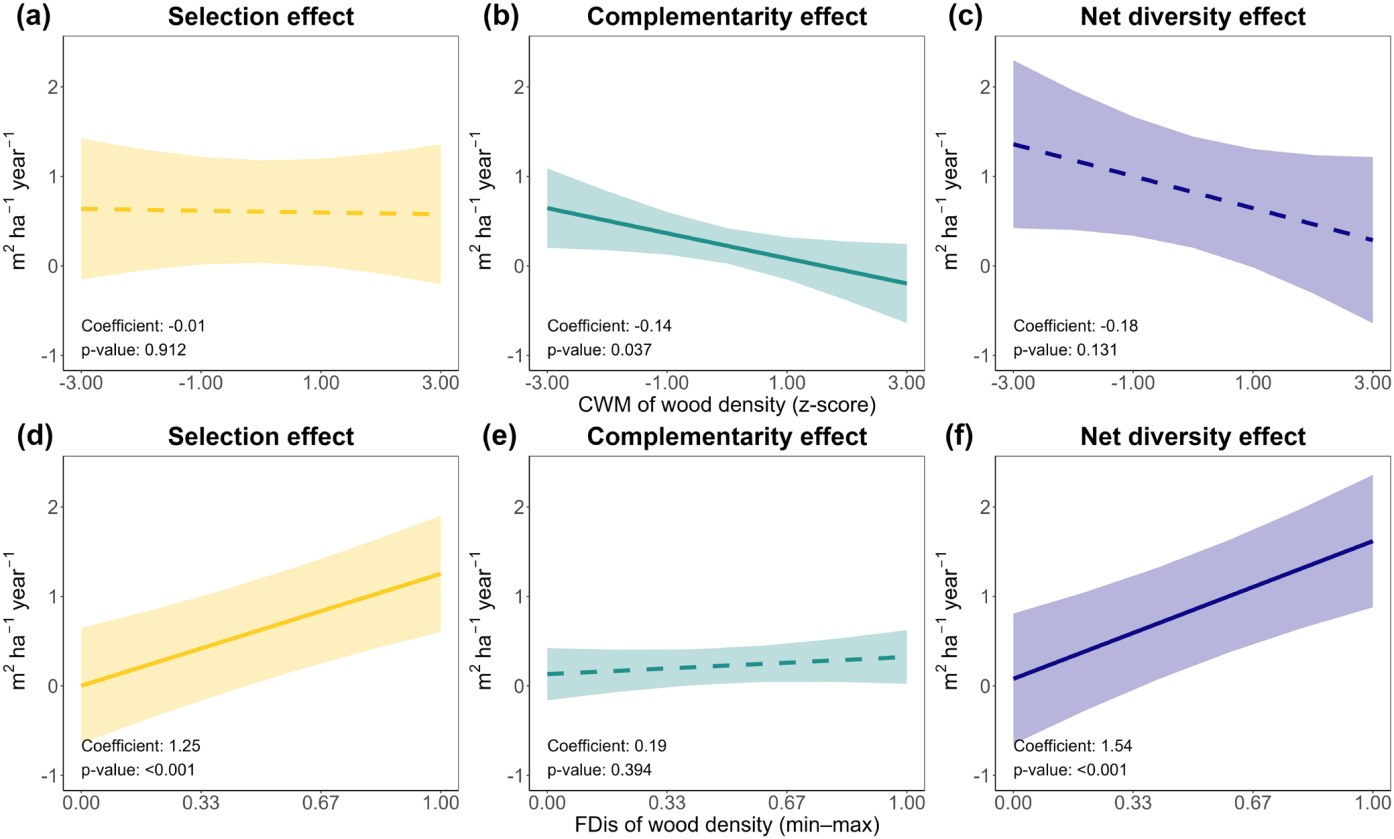
The relationship of selection (a, d; yellow), complementarity (b, e; green) and net diversity (c, f; blue) effects (m^2^ ha^−1^ year^−1^) with community weighted means (CWMs; a–c) and functional diversity (FDis; d–f) of wood density (WD) across 20 experiments. CWMs were *z*‐score standardized and FDis values were min–max standardized prior to analysis. Solid lines show the fitted values for variables that had a significant relationship (dashed lines for non‐significant) (Tables S5–S7). The shaded areas represent a 95% confidence interval. Illustrations showing individual data points can be found in Figure S4.

Overall, the net diversity effect on stand productivity was quantified as 1.05 m^2^ ha^−1^ year^−1^, with the selection effect contributing the majority at 77% (0.81 m^2^ ha^−1^ year^−1^), while the complementarity effect accounted for 23% (0.24 m^2^ ha^−1^ year^−1^). FDis_WD_ was the only significant predictor for both selection and net diversity effect models, showing a positive correlation with coefficients of 1.3 (*p* < 0.001) and 1.5 (*p* < 0.001), respectively (Figure 4d,f; Tables S5–S7). These results indicate that when mixtures were comprised of species with greater WD diversity, productive species made larger gains (a positive selection effect), driving an overall increase in productivity observed in mixed stands compared to monocultures (a positive net diversity effect). Additionally, CWM_WD_ was the sole significant predictor for the complementarity effect, with a coefficient of −0.14 (*p* < 0.05) (Figure 4b; Table S7), indicating that a lower average WD made a small additional contribution to overall productivity via the complementarity effect.

### 3.4 Species With More Acquisitive Traits Achieve Higher Productivity in Mixtures Than Species With More Conservative Traits

**FIGURE 5:**
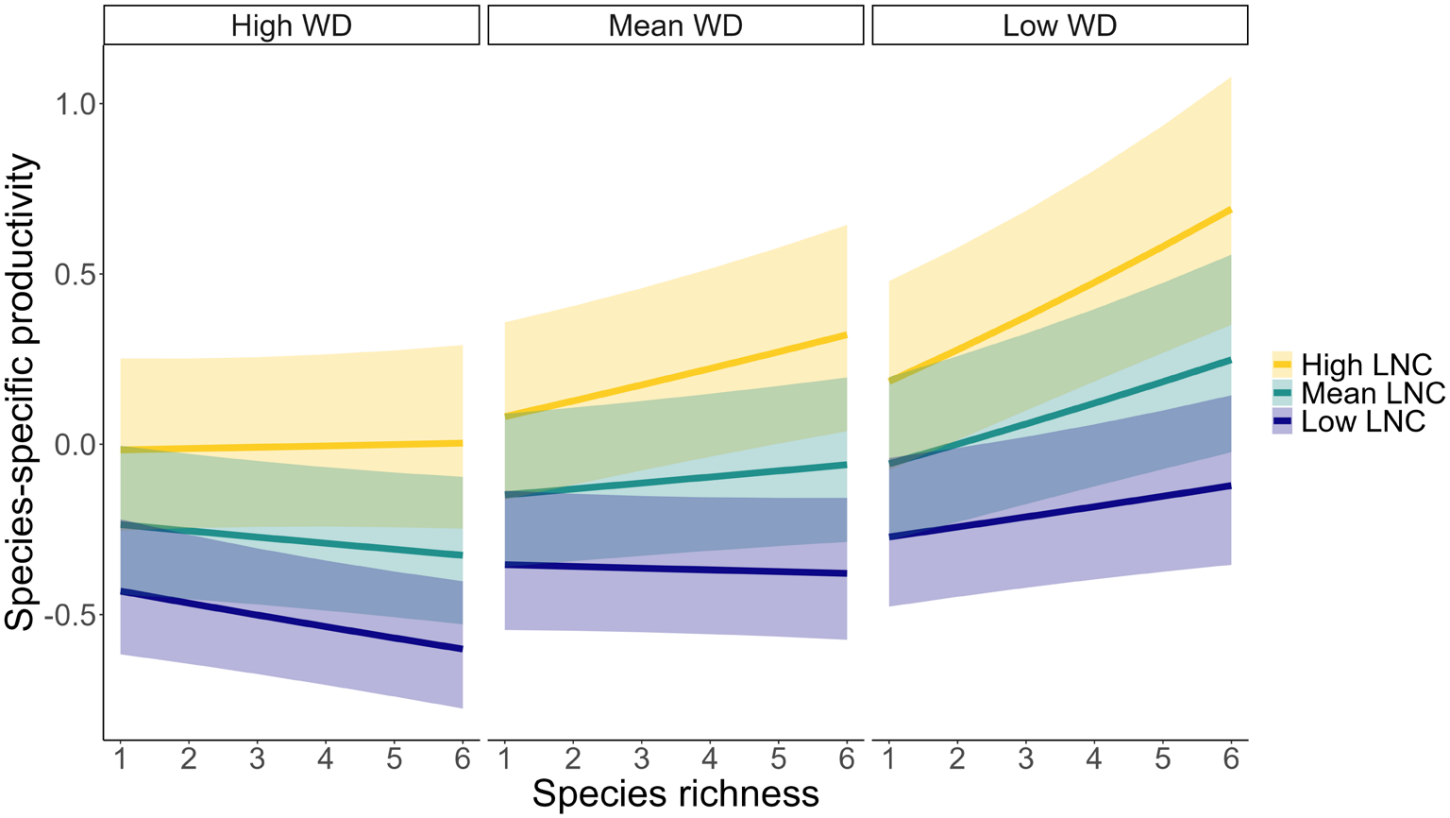
Relationship between species‐specific productivity and species richness across a gradient of more acquisitive species (low wood density [WD] and high leaf Nitrogen content [LNC]) to more conservative species (high WD and low LNC) across 20 experiments. High and low WD and LNC refer to values that are one standard deviation above and below the mean, respectively. Species‐specific productivity corresponds to annual basal area increment, standardized and log‐transformed before analysis. Both WD and LNC were *z*‐score standardized across experiments. Fitted values were back‐transformed from a logarithmic scale prior to illustration, and the shaded areas show the 95% confidence interval for the fitted model. An illustration showing individual data points can be found in Figure S5.

Significant main effects of species richness (0.01, *p* < 0.05) and LNC (0.10, *p* < 0.001) were exhibited on standardized species‐specific productivity on the log‐transformed scale (Figure 5; Table S8). The interaction between species richness and WD (−0.02, *p* < 0.001), and between species richness and LNC (0.01, *p* < 0.01), indicates that the positive effects of species richness were more pronounced for species with higher LNC and lower WD. Across species richness levels, the slopes varied from −0.03 for species with high WD and low LNC to 0.02 for species with mean WD and LNC, and up to 0.10 for species with low WD and high LNC. Additionally, species‐specific productivity became more variable with increasing species richness, with residual variance increasing by 0.07 for each additional species (*p* < 0.001, see Table S8).

## 4 Discussion

Our set of diversity experiments, distributed across the globe, demonstrate that tree species richness enhances stand productivity while reducing its variability. Significantly extending previous analyses (e.g., Belluau et al. 2021; Zheng et al. 2024), we show that this trend is underpinned by the key role played by acquisitive species, especially when combined with more conservative species. We do not observe a significant interaction between species richness and biome, suggesting that our findings are generalizable across a wide geographical range. However, substantial differences between individual experiments suggest that these effects are not uniform, necessitating consideration of site‐specific factors when establishing mixed‐species stands.

Across the gradient of six species, there were indications that the increase was beginning to saturate beyond four to five species in these young stands. These findings align with Gamfeldt et al. (2013), who found that five‐species mixtures produced, on average, 54% more biomass than monocultures. Since other studies (sometimes including longer time periods) have observed even greater productivity increases in more diverse forests, both as planted forests and in species‐rich natural forests (Dolezal et al. 2024; Dormann et al. 2019; Zhang et al. 2012), our results corroborate our first hypothesis and highlight the potential of mixed‐species forestry as a strategy for enhancing productivity and increasing carbon stocks in woody biomass.

Positive effects of species diversity on productivity are often attributed to niche differentiation through functional diversity (Forrester and Bauhus 2016)—a framework supported by our findings and partially corroborating our second hypothesis. Notably, selection effects accounted for 77% of the net diversity effect, compared to 23% for complementarity effects, indicating that globally, high‐performing species can be more important drivers of productivity in young mixed stands than complementarity per se. This finding confirms previous observations made at a less extensive gradient in forest types (Baeten et al. 2019; Tobner et al. 2016). However, studies that have examined the temporal dynamics of diversity–productivity relationships suggest that, as stands mature, complementarity effects tend to increase relative to selection effects (Reich et al. 2012; Urgoiti et al. 2022). Overall, positive diversity effects tend to strengthen over time (Schnabel et al. 2019; Jucker et al. 2020), potentially driven by trait‐dependent shifts that promote species overyielding (Bongers et al. 2021; Zheng et al. 2024). We observed that higher FDis_WD_ amplified selection effects, while lower CWM_WD_ was associated with stronger complementarity effects, consistent with our third hypothesis. These findings suggest that the productivity of young mixed‐species stands is more strongly influenced by individual high‐performing species than by synergistic interactions among species. Importantly, a greater FDis_WD_ within mixed communities reduces functional overlap along many functional axes (Chave et al. 2009; Swenson and Enquist 2007), potentially enabling dominant species to thrive without facing strong resource competition (Kunstler et al. 2012) or having total stand growth reduced by the mortality or poor performance of less competitive species (Chesson 2000). It appears then that stand diversification can be optimized by strategically mixing species that both benefit from and are resilient to the effects of selective processes.

Despite these insights, identifying which species perform well in mixtures using a trait‐based approach remains a challenge (Baeten et al. 2019). Zheng et al. (2024) analyzed data from 65 grassland and forest biodiversity experiments and found that acquisitive species were associated with early overyielding in mixtures. Consistent with our fourth hypothesis, species with higher LNC and lower WD benefited most from mixing, aligning with an acquisitive resource‐use strategy (Poorter and Bongers 2006; Wright et al. 2010). This trait profile may enable species to better exploit the greater niche differentiation in stands with more diverse neighbors. Notably, species with more conservative traits were slightly disadvantaged in mixtures, suggesting that the success of more acquisitive species from species mixing is not necessarily matched by a comparable reduction in growth of more conservative species. This dominance of acquisitive species in mixtures may change as the stands mature, with more conservative, late‐successional species gaining prominence in later stages of stand development (Koricheva et al. 2025; Lohbeck et al. 2013). Our findings align with MacLaren et al. (2023), who reported reduced competitive interactions when mixing species with divergent resource economic strategies in an intercropping system. Our results position WD and LNC as key functional traits for estimating species performance in mixed stands during early growth stages, offering practical tools for managers aiming to maximize stand productivity in this growth phase through informed species selection.

Structural diversity is often proposed as a positive mediator of the species richness–productivity relationship both in planted and natural systems (Dolezal et al. 2020), typically through increased light interception (Rissanen et al. 2019; Williams et al. 2021), though our findings challenge this expectation. We observed that structural diversity had an overall negative effect on productivity, contrasting with our second hypothesis and with previous studies in individual experiments (Fahey et al. 2025; Ray et al. 2023; Schnabel et al. 2019). Different metrics used to quantify structural diversity may capture different aspects of structural diversity responding to different drivers; for example, larger individual trees would make a greater contribution to absolute measures of structural diversity (e.g., LiDAR‐based canopy density metrics), whereas relative metrics, such as the Gini coefficient used in this study, would give equal weight to trees that are performing poorly due to competition. This could explain why structural diversity was associated with a negative effect on growth in monocultures and low‐diversity mixtures in this study; in these stands, the Gini coefficient could be capturing heterogeneity resulting from suppression due to intraspecific competition (Luu et al. 2013; Urgoiti et al. 2023). In contrast, in diverse stands, height differences among trees are more likely to reflect reduced functional overlap (Forrester and Bauhus 2016). Further investigation is required, but such differences between metrics could explain why some studies find positive effects of structural diversity, while other studies agree with the negative relationship observed in this study (Liang et al. 2007; Pretzsch and Hilmers 2024). However, effects of structural diversity may also be highly context‐specific. For example, Ray et al. (2023) found only a marginally positive correlation between structural complexity and light interception, and their study suggested that high light interception is a prerequisite for structural complexity to positively influence productivity in young tree stands. In our study, both species richness and functional diversity appear to moderate the relationship between structural diversity and productivity, suggesting that optimizing functional diversity may mitigate the drawbacks of structural diversity in mixed‐species stands.

This study demonstrates that functional diversity drives the positive relationship between tree diversity and productivity in young stands, while structural diversity exerts a more complex, context‐dependent influence. By linking species traits such as WD and LNC to diversity effects, our findings provide a framework for optimizing tree mixtures to increase both selection and complementarity effects in young stands. Specifically, our analyses partitioning productivity into the complementarity and selection effects, and exploring the effect of community functional composition, suggest that functional diversity in WD in particular is important to attain a mixture of conservative (high WD) species and acquisitive (low WD) species. When mixed, conservative species do not seem to be very impacted by the presence of acquisitive species, while acquisitive species increase productivity because their conservative neighbors do not compete as strongly for resources as their acquisitive conspecifics when planted in monocultures.

We found increased stand productivity at higher species diversity across broad spatial scales. This finding supports the incorporation of species mixtures into future management strategies to store carbon in woody biomass for some decades (in forests and long‐lived wood products) and to substitute fossil carbon–based products or provide bioenergy. Tree diversity can also promote adaptation to climate change, for example, through increased stability in the face of both biotic and abiotic stress and disturbances, including drought (Blondeel et al. 2024; Schnabel et al. 2021; Steckel et al. 2020) and herbivory (Huuskonen et al. 2021; Jactel et al. 2017), which are projected to become more frequent and severe under ongoing global change (Jactel et al. 2019; IPCC 2021). Future research on forest diversity–productivity relationships could explore more complex species assemblages, environmental context–dependency of diversity–productivity relationships (e.g., Ratcliffe et al. 2017), as well as mature, heterogeneous stands where the influence of diversity may be more pronounced. By advancing the understanding of diversity–productivity relationships, this study contributes to leveraging tree diversity in forest management for both ecological and economic objectives.

## Author Contributions

**Joel Jensen:** conceptualization, data curation, formal analysis, investigation, methodology, project administration, software, validation, visualization, writing – original draft, writing – review and editing. **Haben Blondeel:** conceptualization, data curation, investigation, methodology, supervision, writing – review and editing. **Joannès Guillemot:** funding acquisition, investigation, project administration, writing – review and editing. **Florian Schnabel:** conceptualization, investigation, methodology, project administration, supervision, writing – review and editing. **Hernán Serrano‐León:** conceptualization, data curation, investigation, writing – review and editing. **Harald Auge:** investigation, writing – review and editing. **Lander Baeten:** investigation, writing – review and editing. **Nadia Barsoum:** investigation, writing – review and editing. **Jürgen Bauhus:** investigation, writing – review and editing. **Christel Baum:** data curation, supervision, writing – review and editing. **Raimundo Bermudez:** investigation, writing – review and editing. **Friderike Beyer:** data curation, investigation, writing – review and editing. **Pedro Henrique Santin Brancalion:** funding acquisition, investigation, methodology, writing – review and editing. **Jeannine Cavender‐Bares:** investigation, writing – review and editing. **Nico Eisenhauer:** funding acquisition, investigation, project administration, resources, writing – review and editing. **Adam Felton:** conceptualization, writing – review and editing. **Olga Ferlian:** investigation, writing – review and editing. **Sebastian Fiedler:** data curation, investigation, writing – review and editing. **Tobias Gebauer:** investigation, writing – review and editing. **Douglas Godbold:** investigation, writing – review and editing. **Peter Hajek:** data curation, investigation, writing – review and editing. **Jefferson S. Hall:** investigation, writing – review and editing. **Dirk Hölscher:** funding acquisition, investigation, project administration, writing – review and editing. **Hervé Jactel:** investigation, writing – review and editing. **Holger Kreft:** funding acquisition, investigation, project administration, writing – review and editing. **Cathleen Lapadat:** investigation, writing – review and editing. **Chloe MacLaren:** conceptualization, formal analysis, writing – review and editing. **Nicolas Martin:** investigation, writing – review and editing. **Céline Meredieu:** investigation, writing – review and editing. **Simone Mereu:** investigation, writing – review and editing. **Christian Messier:** project administration, writing – review and editing. **Rebecca A Montgomery:** investigation, writing – review and editing. **Bart Muys:** investigation, writing – review and editing. **Charles A. Nock:** investigation, writing – review and editing. **John D Parker:** investigation, writing – review and editing. **William C. Parker:** investigation, writing – review and editing. **Gustavo Paterno:** data curation, investigation, project administration, writing – review and editing. **Michael Perring:** data curation, investigation, writing – review and editing. **Quentin Ponette:** data curation, funding acquisition, investigation, writing – review and editing. **Catherine Potvin:** investigation, writing – review and editing. **Peter B Reich:** investigation, writing – review and editing. **James Rentch:** investigation, writing – review and editing. **Boris Rewald:** investigation, writing – review and editing. **Agnès Robin:** investigation, writing – review and editing. **Hans Sandén:** investigation, writing – review and editing. **Michael Scherer‐Lorenzen:** investigation, writing – review and editing. **Katherine Sinacore:** investigation, writing – review and editing. **Rachel Standish:** data curation, investigation, project administration, resources, writing – review and editing. **Artur Stefanski:** investigation, writing – review and editing. **Kris Verheyen:** investigation, writing – review and editing. **Laura Williams:** data curation, investigation, writing – review and editing. **Martin Weih:** conceptualization, supervision, writing – review and editing.

## Conflicts of Interest

The authors declare no conflicts of interest.

## Supporting information


**Data S1:** gcb70493‐sup‐0001‐DataS1.zip.

## Data Availability

The data that support the findings of this study are openly available in Dryad at https://doi.org/10.5061/dryad.dz08kps9x.
