# Supplementary material for: Diversity in Resource Use Strategies Promotes Productivity in Young Planted Tree Species Mixtures
Source: Glob Chang Biol. 2025 Sep 26;31(9):e70493. doi: 10.1111/gcb.70493 (PMC12475536; doi:10.1111/gcb.70493)
Supplement: Supplementary file 1 — Data S1: gcb70493‐sup‐0001‐DataS1.zip. [file GCB-31-e70493-s001.zip › gcb70493-sup-0001-Supinfo.docx]

**Supplementary materials**

**Experiment-specific considerations**

For the ECOLINK-Uppsala experiment, which operates on three-year harvest cycles, productivity was calculated as the total basal area increment for the final year of each cycle per plot. For BIOTREE Kaltenborn-Species, productivity was calculated by multiplying the basal area increment of each species by scaling factors for (1) the number of patches per subplot, (2) conversion from inventory rows to subplots, and (3) per-hectare scaling. For experiments without true control treatments (i.e. plots where species diversity was the sole experimentally manipulated factor), we used the treatment most similar to natural conditions (e.g. in IDENT SSM where the treatments were irrigation or rainfall exclusion, rainfall exclusion best aligning with natural circumstances. The Ridgefield experiment did not have all species replicated across all species richness levels and was therefore included only in the analysis between species richness and stand productivity (equation 3, Figure 2), as this analysis focuses on the richness gradient rather than species-specific effects. For *Q. pagoda* in the BiodiversiTREE_SERC experiment, there were no studies in the TRY database for leaf nitrogen content (LNC) or wood density (WD), and only a single study for specific leaf area (SLA). Instead, we substituted values from its current sister species, and formerly parent species, *Q. falcata* (N=5-101 studies per metric).


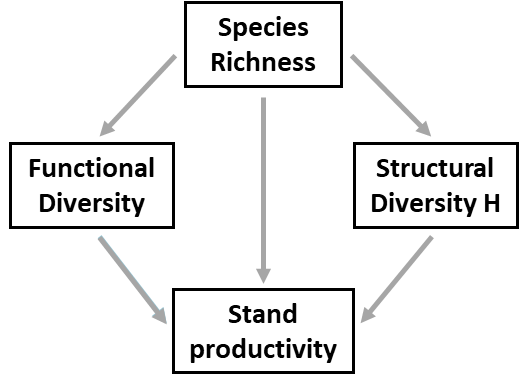


Figure S1. Conceptual structural equation model, before any interactions were included, illustrating direct and indirect links between species richness and standardized stand productivity through functional and structural diversity.

Table S4. Coefficients, standard errors (SE), z-values, and p-values from a regression analysis of the relationship between species richness and standardized stand productivity (m² ha⁻¹ year⁻¹). The dispersion model captures how variability, expressed as the residual standard deviation, changes with increasing species richness.

| **Predictor** | **Coefficient** | **SE** | **z-value** | **p-value** | |
| --- | --- | --- | --- | --- | --- |
| **Conditional model** |  |  |  |  | |
| Intercept | -0.49 | 0.15 | -3.4 | <0.001 | *** |
| Species richness | 0.31 | 0.11 | 2.9 | <0.01 | ** |
| Species richness^2 | -0.03 | 0.02 | -2.0 | <0.05 | * |
| **Dispersion model** |  |  |  |  |  |
| Intercept | -0.68 | 0.08 | -8.4 | <0.001 | *** |
| Species richness | -0.07 | 0.03 | -2.3 | <0.05 | * |


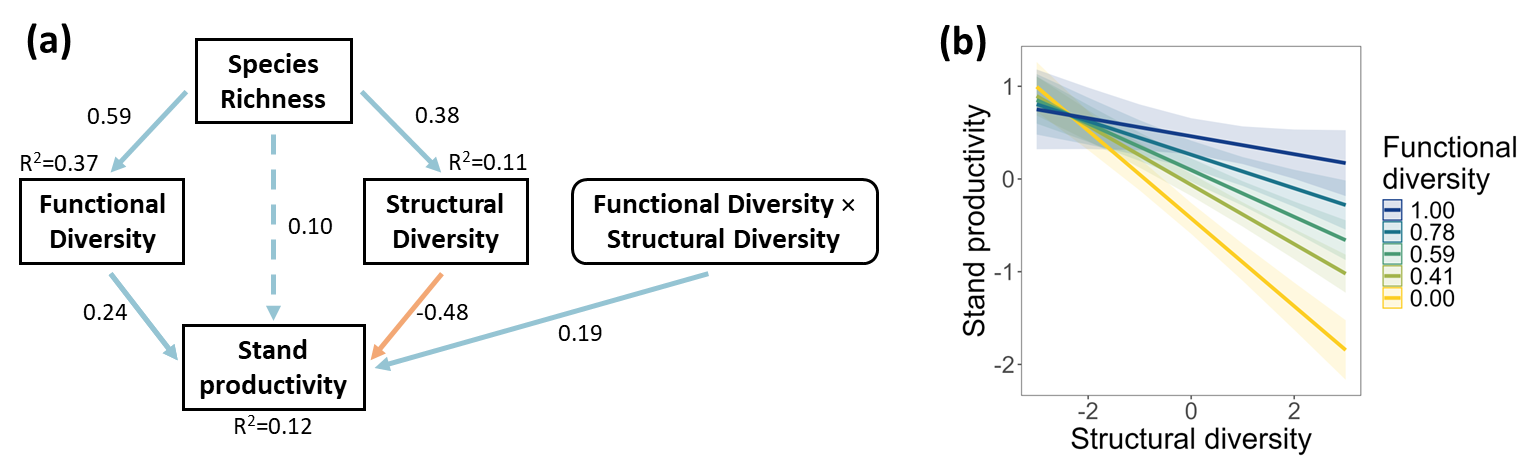


Figure S2. Structural equation model illustrating direct and indirect links between species richness and standardized stand productivity across 16 experiments (a) and the modeled interaction between functional diversity and structural diversity on stand productivity (b). The model displays standardized path coefficients for each pathway, which indicates how many standard deviations the response variables change for every 1 standard deviation change in the predictor. Marginal R² values are shown for each endogenous variable. Blue pathways indicate positive correlations, while orange pathways indicate negative correlations. Significant pathways (p < 0.05) are shown with solid lines. The Fisher´s C statistic for the model was 1.063 with a p-value of 0.59, indicating an adequate fit.


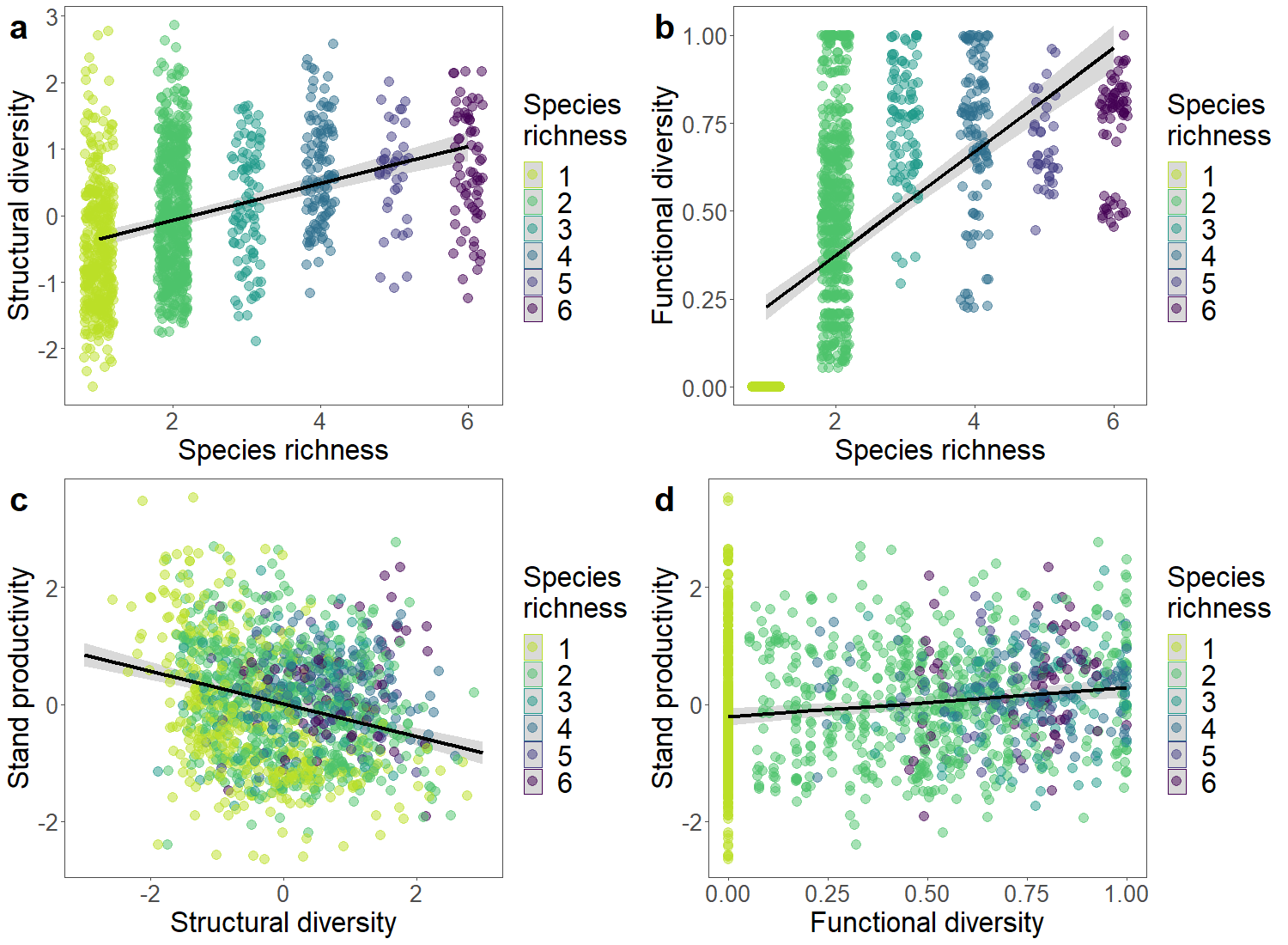


Figure S3. Figures represent the bivariate relationships between variables in the structural equation model illustrated in Figure 2a and are based on the same model structure (equations 4-6). The figures show (a) significant increases in structural diversity with increasing species richness (p < 0.001), (b) significant increases in functional diversity of SLA, LNC and WD with increasing species richness (p < 0.001), (c) significant decreases in stand productivity with increasing structural diversity, and (d) significant increases in stand productivity with increasing functional diversity of SLA, LNC and WD (p < 0.001). Data points are colored based on a species richness gradient of 1-6 species. Shaded areas represent a 95% confidence interval.


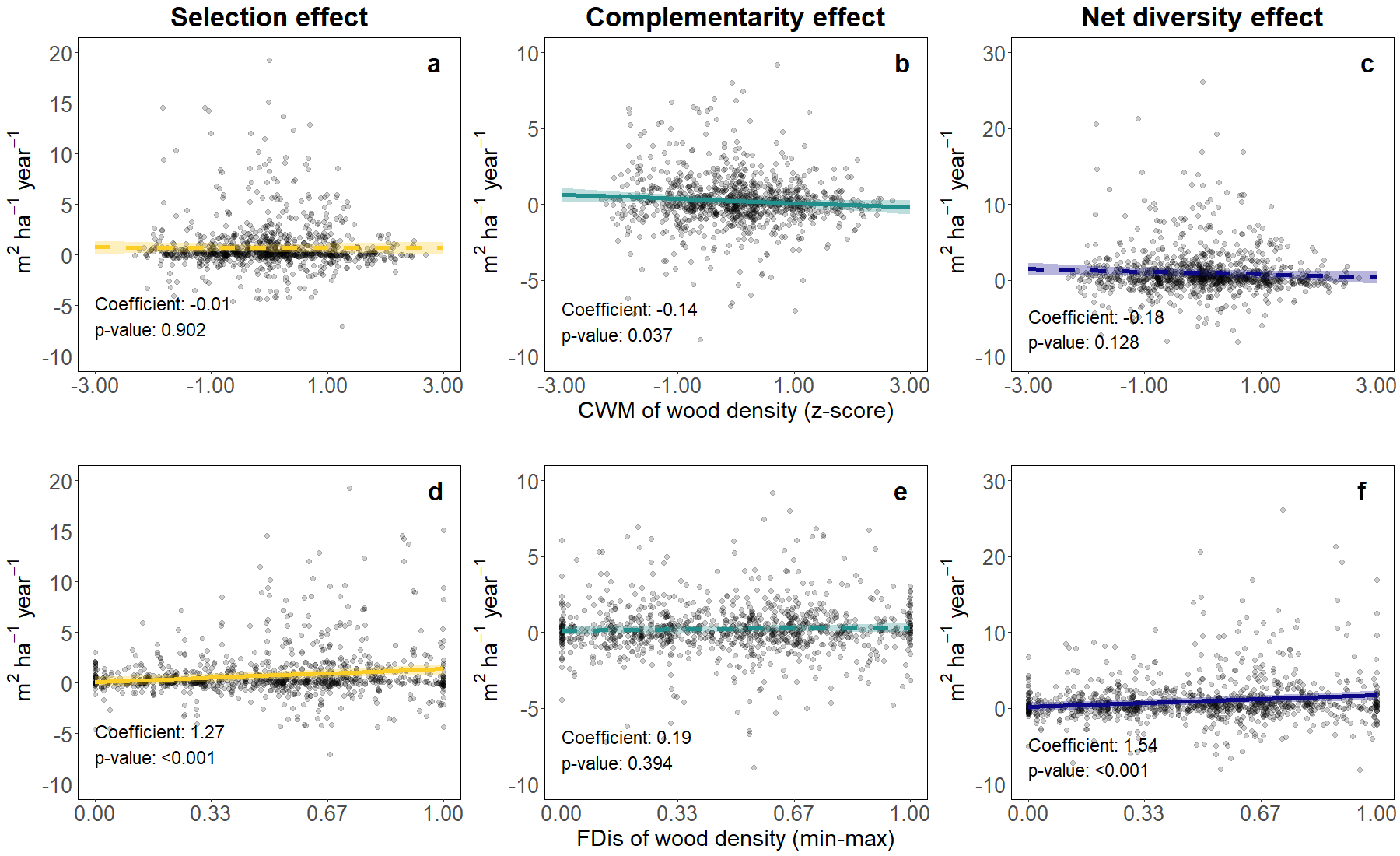


Figure S4. Selection- (a & d; yellow), complementarity- (b & e; green), and net diversity (c & f; blue) effects over community weighted means (CWMs; a-c) and functional diversity (FDis; d-f) of~~,~~ wood density (WD) across 20 experiments. CWMs were z-score standardized and FDis values were min-max standardized prior to analysis. Solid lines show the fitted values for variables that had a significant relationship (dashed lines for non-significant) (Tables S5-S7). The shaded areas represent a 95% confidence interval.

Table S5. Summary of parameter estimates, standard errors (SE), z-values, and p-values for predictors of the selection effect, including community weighted means (CWMs) and functional diversity (FDis) for wood density (WD), and leaf nitrogen content (LNC). The dispersion model captures how variability, expressed as the residual standard deviation, changes with increasing species richness.

| **Predictor** | **Coefficient** | **SE** | **z-value** | **p-value** | |
| --- | --- | --- | --- | --- | --- |
| **Conditional model** |  |  |  |  | |
| Intercept | 0.14 | 0.28 | 0.04 | 0.68 |  |
| CWM_WD_ | -0.01 | 0.09 | -0.12 | 0.90 |  |
| CWM_LNC_ | -0.05 | 0.09 | -0.6 | 0.54 |  |
| FDis_WD_ | 1.25 | 0.31 | 4.11 | <0.001 | *** |
| FDis_LNC_ | 0.31 | 0.30 | 0.84 | 0.29 |  |
| **Dispersion model** |  |  |  |  |  |
| Intercept | -0.08 | 0.06 | -1.2 | 0.25 |  |
| Species richness | 0.09 | 0.02 | 4.3 | <0.001 | *** |

Table S6. Summary of parameter estimates, standard errors (SE), z-values, and p-values for predictors of the complementarity effect, including community weighted means (CWMs) and functional diversity (FDis) for wood density (WD), and leaf nitrogen content (LNC). The dispersion model captures how variability, expressed as the residual standard deviation, changes with increasing species richness.

| **Predictor** | **Coefficient** | **SE** | **z-value** | **p-value** | |
| --- | --- | --- | --- | --- | --- |
| **Conditional model** |  |  |  |  | |
| Intercept | 0.02 | 0.17 | 0.15 | 0.88 |  |
| CWM_WD_ | -0.14 | 0.07 | -2.10 | 0.04 | * |
| CWM_LNC_ | 0.06 | 0.07 | 0.90 | 0.37 |  |
| FDis_WD_ | 0.19 | 0.23 | 0.85 | 0.39 |  |
| FDis_LNC_ | 0.23 | 0.21 | 1.09 | 0.27 |  |
| **Dispersion model** |  |  |  |  |  |
| Intercept | 0.46 | 0.06 | 7.5 | <0.001 | *** |
| Species richness | -0.01 | 0.02 | -0.3 | 0.78 |  |

Table S7. Summary of parameter estimates, standard errors (SE), z-values, and p-values for predictors of the net diversity effect, including community weighted means (CWMs) and functional diversity (FDis) for wood density (WD), and leaf nitrogen content (LNC). The dispersion model captures how variability, expressed as the residual standard deviation, changes with increasing species richness.

| **Predictor** | **Coefficient** | **SE** | **z-value** | **p-value** | |
| --- | --- | --- | --- | --- | --- |
| **Conditional model** |  |  |  |  | |
| Intercept | -0.22 | 0.40 | -0.54 | 0.59 |  |
| CWM_WD_ | -0.18 | 0.12 | -1.51 | 0.13 |  |
| CWM_LNC_ | -0.01 | 0.11 | -0.11 | 0.91 |  |
| FDis_WD_ | 1.54 | 0.40 | 3.85 | <0.001 | *** |
| FDis_LNC_ | 0.64 | 0.38 | 1.68 | 0.09 |  |
| **Dispersion model** |  |  |  |  |  |
| Intercept | 0.53 | 0.06 | 7.5 | <0.001 | *** |
| Species richness | 0.08 | 0.02 | 3.7 | <0.001 | *** |


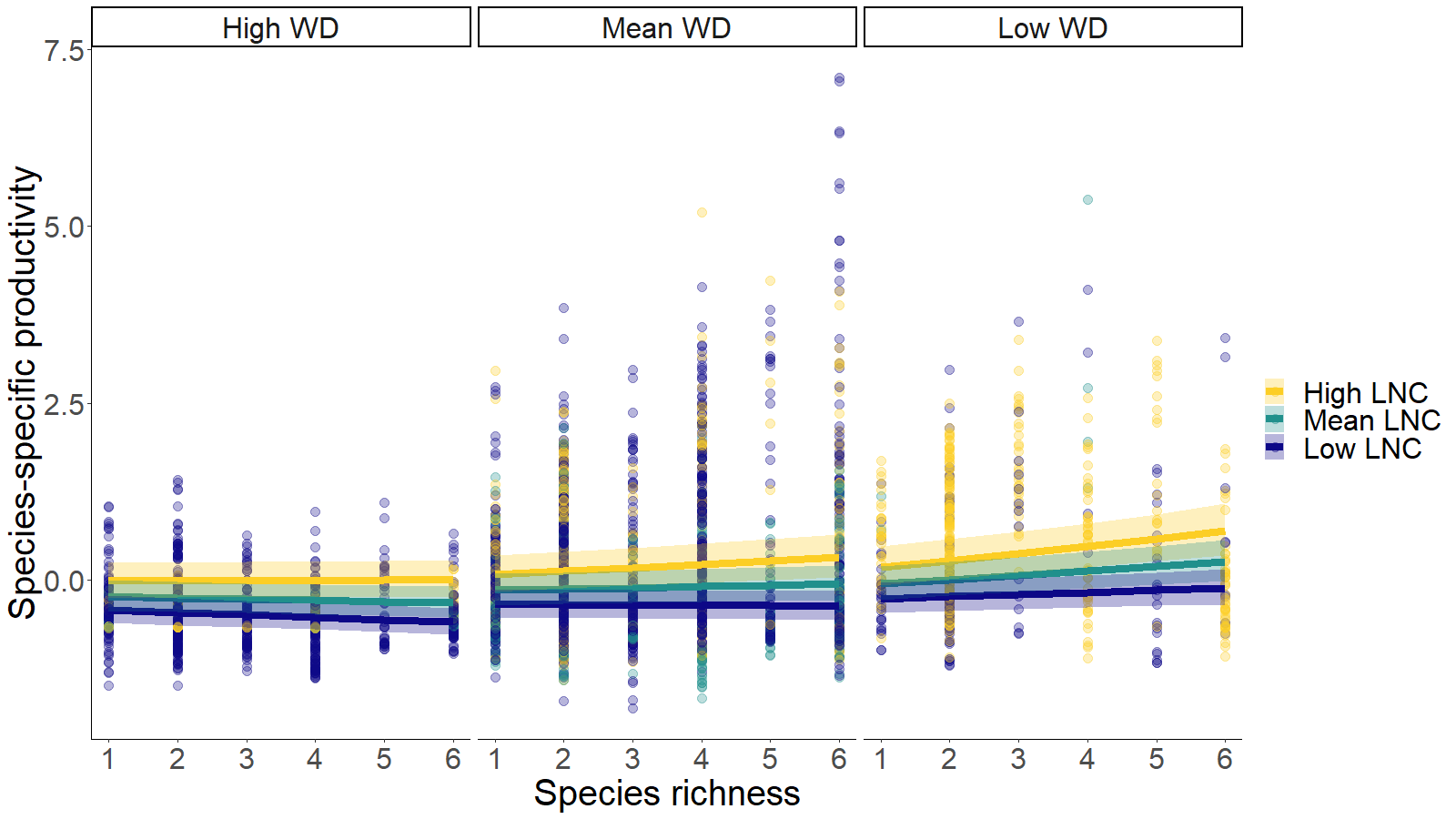
Figure S5. Relationship between species-specific productivity and species richness across a gradient of more acquisitive species (low wood density (WD) and high leaf nitrogen content (LNC)) to more conservative species (high WD and low LNC) across 20 experiments. High and low WD and LNC refer to values that are one standard deviation above and below the mean, respectively. Species-specific productivity corresponds to annual basal area increment, standardized and log-transformed before analysis. Both WD and LNC were z-score standardized across experiments. Fitted values were back-transformed from a logarithmic scale prior to illustration and the shaded areas show a 95% confidence interval for the fitted model.

Table S8. Model parameters for the analysis of species-specific productivity, examining species richness, wood density (WD), and leaf nitrogen content (LNC) as predictors. Species-specific productivity corresponds to annual basal area increment (m^2^ ha^-1^ year^-1^). Coefficients, standard errors (SE), z-values, and p-values are reported for main and interaction effects. The dispersion model captures how variability, expressed as the residual standard deviation, changes with increasing species richness.

| **Predictor** | **Coefficient** | **SE** | **z-value** | **p-value** | |
| --- | --- | --- | --- | --- | --- |
| **Conditional model** |  |  |  |  | |
| Intercept | 0.611 | 0.063 | 10 | <0.001 | ******* |
| Species richness | 0.010 | 0.005 | 2 | <0.05 | * |
| WD | -0.028 | 0.015 | -2 | 0.080 |  |
| LNC | 0.105 | 0.017 | 6 | <0.001 | ******* |
| Species richness : WD | -0.020 | 0.005 | -4 | <0.001 | ******* |
| Species richness : LNC | 0.013 | 0.004 | 3 | <0.01 | ** |
| **Dispersion model** |  |  |  |  |  |
| Intercept | -1.21 | 0.03 | -40.7 | <0.001 | *** |
| Species richness | 0.066 | 0.01 | 7.9 | <0.001 | *** |


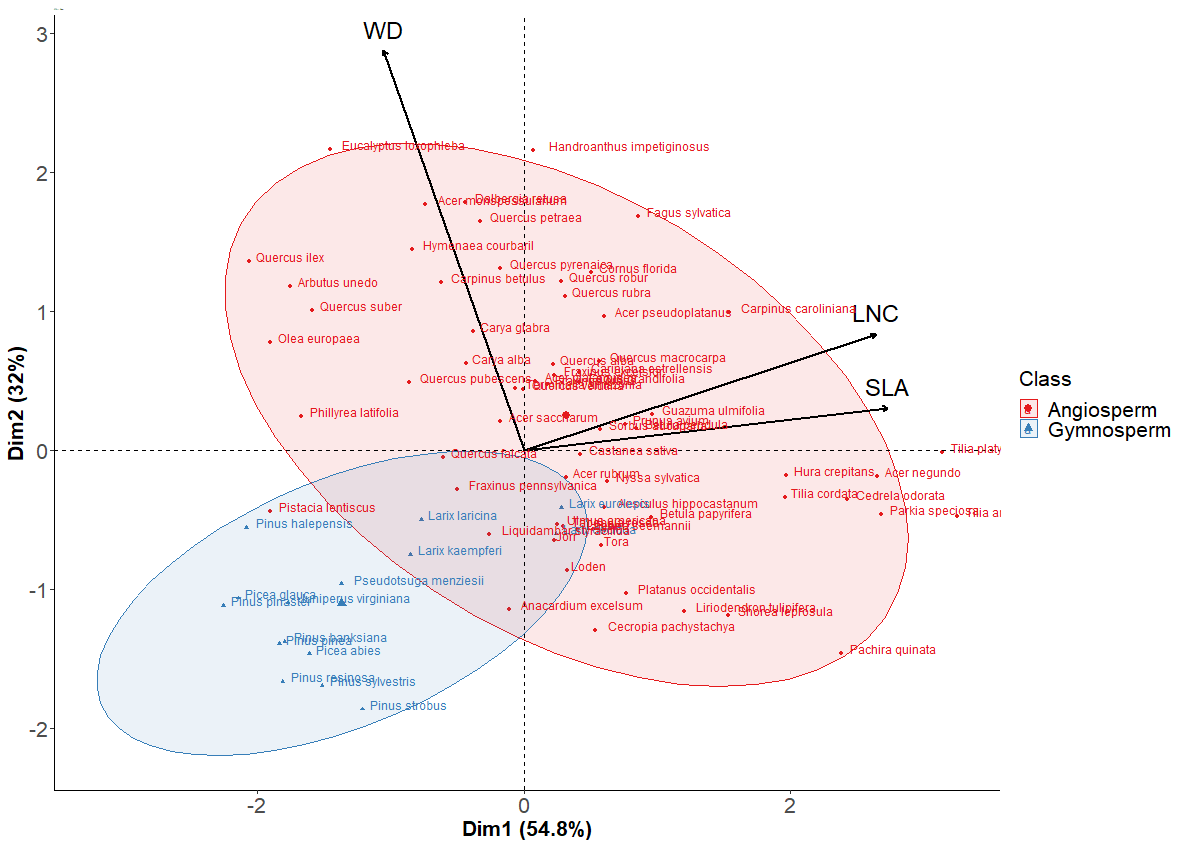
Figure S6. Principal Component Analysis (PCA) of species included in the study, with full data for all traits: specific leaf area (SLA), leaf Nitrogen content (LNC), and wood density (WD). Ellipses represent 90% of the species distribution. Angiosperms are depicted in red, and gymnosperms in blue. Detailed information on traits can be found in Supplementary Table S2.
