# Supplementary material for: Diversity in Resource Use Strategies Promotes Productivity in Young Planted Tree Species Mixtures
Source: Glob Chang Biol. 2025 Sep 26;31(9):e70493. doi: 10.1111/gcb.70493 (PMC12475536; doi:10.1111/gcb.70493)
Supplement: Supplementary file 1 — Data S1: gcb70493‐sup‐0001‐DataS1.zip. [file GCB-31-e70493-s001.zip › Table S1-S3 legends.docx]

**Table S1.** Overview of experiments included in this study, showing biome, location, geographic coordinates, altitude, planting and inventory years, experiment age, species richness levels, species composition, plot size, planting density, measurement heights, sampling proportions, use in the structural equation model, and number of plots.

**Table S2.** Overview of species functional trait data for all species included in this study, specifying angiosperm or gymnosperm status, specific leaf area (SLA), leaf nitrogen content (LNC), and wood density (WD), together with the source of each trait value (site inventory, TRY database, or published literature).

**Table S3.**Overview of variables used in this study, including abbreviations, variable type (indicating whether a variable represents an aspect of productivity, structural diversity, functional identity, functional diversity, or diversity effects), units, mean and standard deviation across experiments for original and transformed values, transformation method, and transformation scale (within or across experiments).
